# Supplementary material for: Association between oral microbiome and seven types of cancers in East Asian population: a two-sample Mendelian randomization analysis
Source: Front Mol Biosci. 2023 Nov 21;10:1327893. doi: 10.3389/fmolb.2023.1327893 (PMC10702768; doi:10.3389/fmolb.2023.1327893)
Supplement: Supplementary file 1 [file DataSheet1.ZIP › Supplementary Materials/MR plots for tongue/tongue═╝/Pancreatic cancer/╥╚╧┘░⌐_╔╕╤í╜ß╣√/pheno.85_to_pancreatic cancer_forest.pdf]

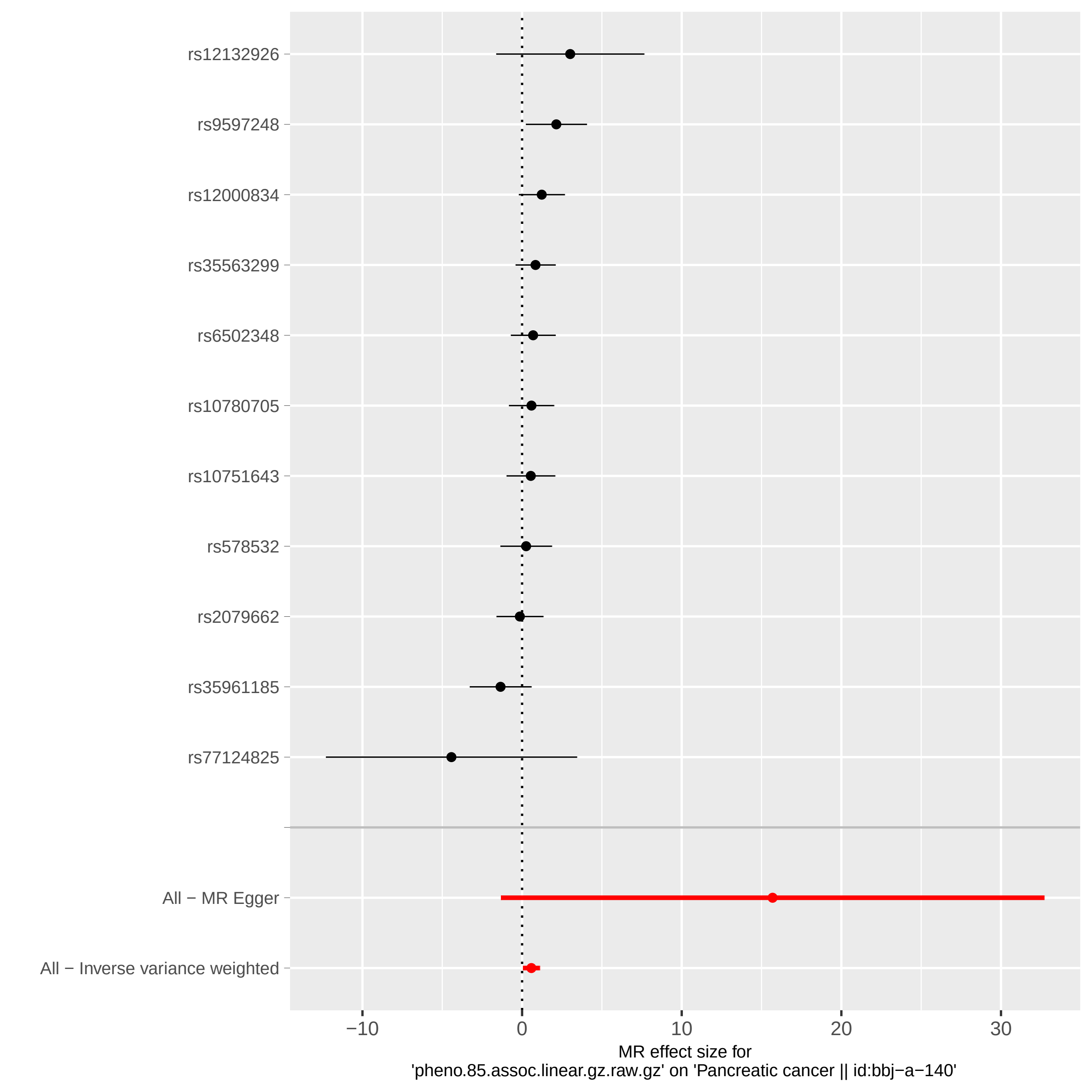
rs12132926
rs9597248
rs12000834
rs35563299
rs6502348
rs10780705
rs10751643
rs578532
rs2079662
rs35961185
rs77124825
All − MR Egger
All − Inverse variance weighted
−10
0
10
20
30
MR effect size for
'pheno.85.assoc.linear.gz.raw.gz' on 'Pancreatic cancer || id:bbj−a−140'
